# Tolerance and decolorization potential of duckweed (*Lemna gibba*) to C.I. Basic Green 4

**DOI:** 10.1038/s41598-021-90369-2

**Published:** 2021-05-25

**Authors:** Hanwant Singh, Shani Raj, Deepak Kumar, Shubhangani Sharma, Upma Bhatt, Hazem M. kalaji, Jacek Wróbel, Vineet Soni

**Affiliations:** 1 Plant Bioenergetics and Biotechnology Laboratory, Department of Botany, Mohanlal Sukhadia University, Udaipur, Rajasthan 313001 India; 2 Department of Plant Physiology, Institute of Biology, Warsaw, University of Life Sciences, Nowoursynowska 159, 02-776 Warsaw, Poland; 3 Department of Bioengineering, West Pomeranian University of Technology in Szczecin, 17 Słowackiego Street, 71-434 Szczecin, Poland

**Keywords:** Biochemistry, Biological techniques, Biophysics, Biotechnology, Plant sciences, Environmental sciences

## Abstract

With growing human culture and industrialization, many pollutants are being introduced into aquatic ecosystems. In recent years, dyes have become a major water pollutant used in the manufacture of paints and other production purposes. In this research, the potential of duckweed (*Lemna gibba*) plant was investigated spectrophotometrically as an obvious bioagent for the biological decolorization of the organic dye C.I. Basic Green 4 (Malachite Green, BG4). Photosynthetic efficiency analysis showed that the photosynthetic apparatus of *L. gibba* is very tolerant to BG4. Significant induction of reactive oxygen species (ROS) scavenging enzymes was observed after 24h of biodecolorization process in *L. gibba* treated with 15 and 30 mg/l BG4. The experimental results showed that *L. gibba* has a strong ability to extract BG4 from contaminated water and the best results were obtained at 25–30°C and pH 8.0. We conclude that duckweed *L. gibba* can be used as a potent decolorization organism for BG4.

## Introduction

With increasing human civilization and industrialization, many pollutants are introduced into the aquatic ecosystem and thus integrated into the food chain, ultimately leading to harmful effects on daily life. Dyes have become a significant water pollutant in recent years due to their use in paint processing and other industrial purposes^1^. Dyes can be divided into two types: natural and synthetic. Natural dyes have the advantage of easy natural precipitation. Nowadays, synthetic dyes dominate in textile industry due to their long shelf life, insensitivity to environmental conditions and wide range of colors^2^. Generally, the colour of dyes in the water is noticeable at concentration of 1 mg/l^3^. It has been reported that the dye concentration in wastewater from textile industry is found 300 mg/l and 80,000 tons of commercial dyes discharge in the wastewater^4^.

Water pollution with dyes has a negative effect on the photosynthesis process of aquatic plants. The presence of the dye in the water reduces the penetration of light into the water (preventing light from reaching the lower layers of the water)^5,6^. Therefore, removal of all pollutants from wastewater is the main goal for mankind^7^. The conventional wastewater treatment technologies such as physical and chemical, are more expensive and introduce other toxic byproducts which are hazardous and required further processing^8,9^. To overcome this problem, there are many biological agents such as bacteria, fungi, yeasts, and algae have been reported to remove or degrade dyes from contaminated water^9,10^. Some plant species have also been reported to have potential to decolorize dyes^11–14^.

Basic Green 4 (BG 4, Malachite Green) is a cationic dye commonly used to dye silk, leather and wool and is also used as a fungicide^15^. Literature describing influence of BG4 on aquatic environments revealed that there is a huge negative impact on various animal species^16^. Leucomalachite a reduced form of BG4 remains long time in edible fish which more toxic for human health. Therefore, it is crucial need to remediate the wastewater^17,18^.

Photosynthesis apparatus, specially photosystem II is one of the key targets of all types of environmental stresses. Chlorophyll fluorescence analysis gives an opportunity to understand the photosynthetic performance of plants under adverse climatic conditions^19^. Oxygen containing chemically-reactive molecules are known as Reactive oxygen species (ROS) *i.e.* superoxide (O_2_^.-^), hydroxyl radical (OH^*^) and hydrogen peroxide (H_2_O_2_) which are produced as a byproduct of reduction metabolism of oxygen and participates in plant development and signal transduction. Increased amount of ROS causes oxidative stress or cell death. Under some adverse environmental circumstances and due to increased level of ROS plants may face oxidative stress^20^.

In the present study, *Lemna gibba* L. was used to examine its biodecolorization potential of organic dye BG4 commonly known as Malachite Green. *L. gibba* is an aquatic macrophyte commonly known as gibbous duckweed, swollen duckweed or fat duckweed. *L. gibba* belongs to the family Lemnaceae and its small size, high multiplication rate, and reduced anatomy makes it a good candidate for phytoremediation^21–23^. To assess the ability of *L. gibba* to decolorize BG4, various parameters such as initial biomass concentration, temperature and pH were implied. Here, we have studied the ROS scavenging enzymes status in *L. gibba* in order to get insights into the role of BG4 dye in bringing on oxidative stress. We examined the change in antioxidant activity by analyses three Reactive Oxygen Species (ROS) scavenging enzymes: superoxide dismutase (SOD; EC.1.15.1.1), catalase (CAT; EC 1.11.1.6), and guaiacol peroxidase (GPOD; EC 1.11.1.7). The repeated batch operation was also performed to determine the reusability of *L. gibba* for dye decolorization. Through the analysis of polyphasic chlorophyll fluorescence induction curves, efforts were made to study the impact of the dye decolorization process on photosystem II (PSII) photochemistry of *L. gibba*.

The aim of this study was to check the potential of duckweed plants (*Lemna gibba*) as a powerful decolorization organism of Basic Green 4 (BG 4) dye, which is commonly used by various industrial sectors.

## Materials and methods

### Plant materials and growth condition

**Figure 1:**
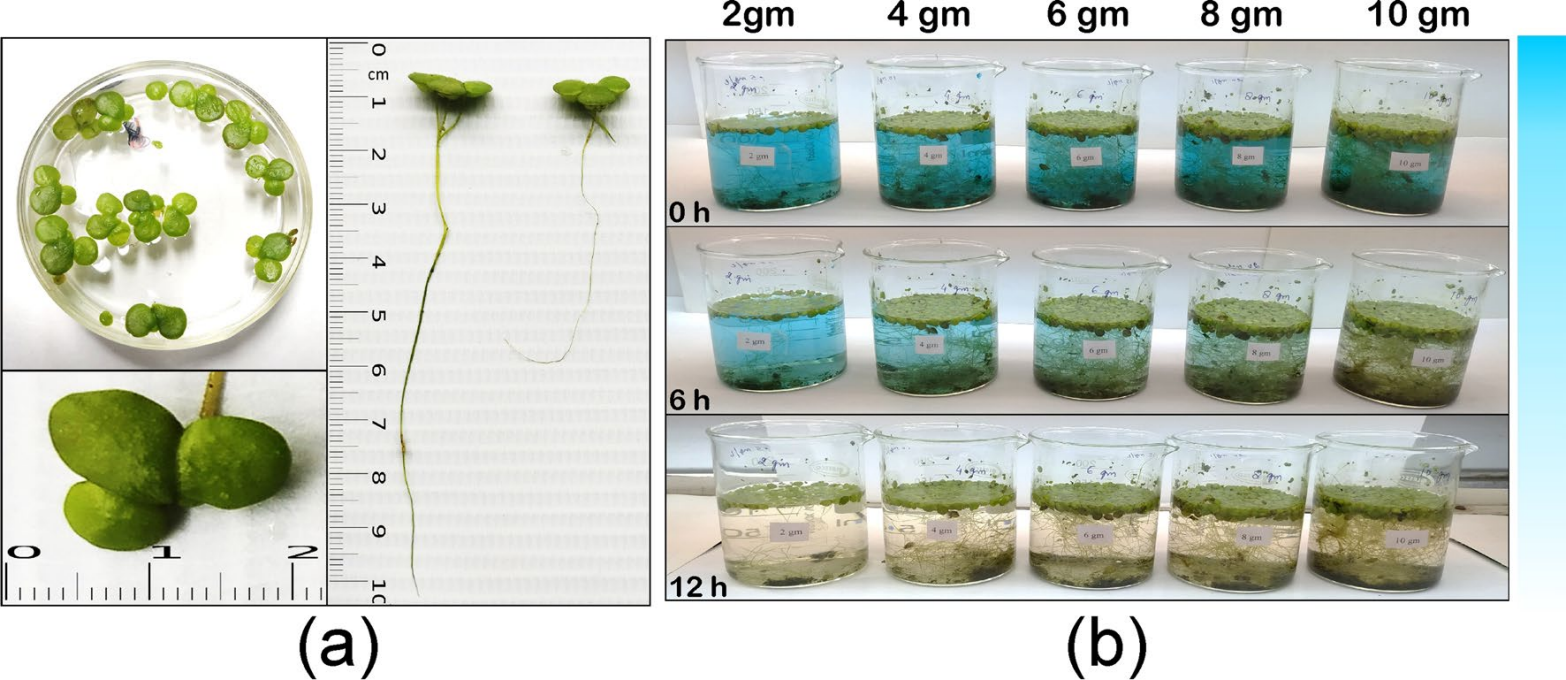
Macroscopic view of fronds and rhizoids of *L. gibba* **(a) **and physical observation of *L. gibba*-mediated decolorization **(b)**.

In the present investigation duckweed, *L. gibba* was used for phytoremediation of triphenylmethane dye BG4. Plant material (Fig. 1a) was collected from the region of Ayad river located at Udaipur, India (24°35′14.97"N, 73°42′38.75"E). Details about water properties and environmental condition of Ayad river are given in Table 1. BG4 was procured from HIMEDIA (India). The plants were rinsed with double distilled water to remove surface contamination and maintained in plastic pond under illumination provided by white fluorescent light with 6500–10,000 lx light irradiance, 14-h photoperiod, and 25/20 °C day/night temperature for three months as a pre-treatment before experiments (as per OECD guidline, 2002)^24^. A full-strength Jacob culture medium was prepared according to the Table 2 and pH maintained at 6.0. The medium was replaced every two months and the circulation was provided with an electric pump.

**Table 1:** Water properties and environmental conditions of sample collection site.

S. No	Parameters	Unit	Range
**Water properties**			
1	Colour		Dark brown
2	pH		7.2–8.1
3	Conductivity	mS/cm	0.35–0.58
4	Total dissolved solids (TDS)	ppm	560–993
5	Dissolved oxygen	ppm	0.11–0.39
6	BOD	ppm	55.3–86
7	COD	ppm	159–216
8	Alkalinity	ppm	298–320
9	Total hardness (As CaCO_3_)	ppm	565–286
10	Sulphate (As SO4)	ppm	75.6–89.6
11	Chloride (As Cl−)	ppm	486–796
12	Fluoride (F−)	ppm	8.4–8.9
13	Silica (As SiO2)	ppm	39–68
14	Sodium	ppm	22.5–187
15	Total organic carbon (TOC)	ppm	11–18
**Environmental conditions**			
1	Temperature	C	20–25
2	Humidity	%	67

**Table 2:** Composition of Jacob’s medium.

Stock solution	Composition	Concentration (g/l)
A	Ca (NO_3_)_2_	60.000
B	MgSO_4_·7H_2_O KNO_3_ KH_2_PO_4_	102.000 100.000 14.000
C	H_3_BO_4_ MnCl_2_·4H_2_O ZnSO_4_·7H_2_O Na_2_MoO_4_·2H_2_O	0.3000 0.3145 0.0356 0.0118
D	CuSO_4_·5H_2_O FeEDTA^a^	0.0125 1.8520

^a^Ethylenediaminetetraacetatic acid. Experiment media were prepared by taking 10 ml aliquot of each of the stock solutions and makeup up to 1 L in a volumetric flask.

### Experiment

#### Photosynthetic performance

##### Fast Chl *a* fluorescence kinetic transient

To determine changes in Chlorophyll (Chl) *a* fluorescence, O-J-I-P transient was recorded after 12 h of dye treatment by Plant Efficiency Analyzer, PEA (*Hansatech Instruments*, Kings Lynn, Norfolk, U.K.). Before the measurements, control and treated plants were adapted to darkness in room for 1 h, and additionally, the measured spots were kept in darkness in the clip for 1 min just before measurement^25^. Fluorescence transients were induced over a leaf area of 4 mm diameter by a red light (peak at 650 nm) of 3000 µmol m^-2^ s^-1^ (sufficient excitation intensity to ensure closure of all PSII RCs to obtain a maximal fluorescence intensity of F_M_) provided by a high intensity LED array of three light-emitting diodes. A total measuring time of one second was used thought out the experiments^19^. Abbreviations, formulas, and definitions of the JIP-test parameters used in the current study are presented in Table 3^26,27^.

**Table 3:** Abbreviations, formulas, and definitions of the JIP-test parameters used in the current study (Strasser et al. 2000, 2004).

Technical fluorescence parameter	Formulas	Definitions
**Data extracted from the recorded fluorescence transient O-J-I-P**		
t_FM_		Time (in ms) to reach the maximal fluorescence intensity FM
Area		Total complementary area between the fluorescence induction curve and F = FM
F_0_	≅ F_50µs_ or ≅ F_20µs_	Minimal fluorescence (all PSII RCs are assumed to be open)
F_V_	≡ F_M_ - F_0_	Maximal variable fluorescence
F_M_ (= F_P_)		Maximal fluorescence, when all PSII RCs are closed
**Specific energy flux**		
ABS/RC	= M_0_ (1/V_J_) (1/_Po_)	Absorption flux (of antenna Chls) per RC
TR_0_/RC	= M_0_ (1/V_J_)	Trapped energy flux (leading to Q_A_ reduction) per RC
ET_0_/ RC	= M_0_ (1/V_J_)_Eo_	Electron transport flux (further than Q_A_^−^) per RC
**Quantum yields and efficiencies**		
_Po_	≡ TR_0_/ABS = [1 - (F_0_/F_M_)]	Maximum quantum yield for primary photochemistry
_Eo_	≡ ET_0_/TR_0_ = (1 - V_J_)	Efficiency/probability for electron transport (ET), i.e. efficiency/probability that an electron moves further than Q_A_^−^
_Eo_	≡ ET_0_/ABS = [1 - (F_0_/F_M_)]_Eo_	Quantum yield for electron transport (ET)
PI_ABS_	≡$\frac{{\gamma }_{RC}}{1-{\gamma }_{RC}}\cdot \frac{{\phi }_{Po}}{1-{\phi }_{Po}}\cdot \frac{{\psi }_{o}}{1-{\psi }_{o}}$	Performance index (potential) for energy conservation from exciton to the reduction of intersystem electron acceptors
RC/ABS	= γ_RC_/(1-γ_RC_ ) = _Po_ (V_J_/ M_0_)	Q_A_-reducing RCs per PSII antenna Chl (reciprocal of ABS/RC)
DI_o_/RC	= (ABS/RC) - (TR0/RC)	Total energy dissipated per reaction center (RC)
*F*_V_/*F*_O_		Ratio of rate constants for photochemical and nonphotochemical use of RC excitation energy
(*dV*/*dt*)_0_	4. (F_300_–*F*_0_) / ( *F*_M_–*F*_0_ )	Maximal rate of the accumulation of the fraction of closed reaction centers
ABS / CS_m_	*F*_*m*_	Absorption flux (of antenna Chls) per RC
**Phenomological energy flux**		
TR_0_ / CS_m_	(Fv/Fm) (ABS/CS_m_)	Trapped energy flux (leading to Q_A_ reduction) per RC
ET_0_ / CS_m_	(Fv/Fm) (1 - Vj) (ABS/CS_m_)	Electron transport flux (further than Q_A_^−^) per RC
DI_0_ / CS_m_	(ABS/CS0) - (TR0/CS_m_)	Total energy dissipated per reaction center (RC)
RC / CS_m_	ϕPo (V_J_ / *M*_0_) *F*_m_	Reaction centre per cross section

##### Specific and phenomenological fluxes

Specific activities of active PSII reaction centre i.e. antenna size of an active PSII (ABS/RC), electron transport flux from QA to PQ per active PSII (ET/RC) and Dissipated energy flux per reaction centre (DI/RC) and phenomenological fluxes i.e. Absorption flux per cross section (ABS/CS), Electron transport flux per cross section (ET/CS) and Dissipated energy flux per cross section (DI/CS) were calculated using following equations of JIP test^19^.

1 $$\frac{ABC}{{RC}} = Mo{ }\cdot{ }\left( \frac{1}{Vj} \right)\cdot{ }\left( {\frac{1}{\phi Po}} \right)$$

2 $$\frac{TR}{{RC}} = Mo{ }\cdot\left( \frac{1}{Vj} \right)$$

3 $$\frac{ET}{{RC}} = Mo \cdot \left[ {1/\frac{{\left( {{\text{F}}2{\text{ms}} - {\text{Fo}}} \right)}}{{\left( {{\text{Fm}} - {\text{Fo}}} \right)}}} \right]\cdot\Psi o$$

4 $$\frac{ABS}{{CS}} = { }Flourescence{ }intensity{ }at{ }50{{\mu s }}\left( {Fo} \right)$$

5 $$\frac{TR}{{CS}} = \phi Po\cdot\left( {ABS/CS} \right)$$

6 $$\frac{ET}{{CS}} = { }\phi Po\cdot\Psi o\cdot\left( {\frac{ABS}{{CS}}} \right)$$

7 $$ET/CS = { }\phi Po\cdot\Psi o\cdot\left( {\frac{ABS}{{CS}}} \right)$$

where Mo is approximated initial slope (in m s^−1^) of the fluorescent transient, calculated as 4 × (F_300_ − F_0_)/(F_M_ − F_0_) and Ψ_0_ is calculated as 1 − V_J_. V_j_ is relative variable fluorescence at the J-step and calculated as (F_2ms_ − F_0_)/(Fm − Fo).

##### Density of active PSII RCs

The concentration of active PSII RCs (RC/CS) was quantified as per the following formula.

8 $$\frac{RC}{CS}= \phi Po\cdot \left[\frac{Vj}{Mo}\right]\cdot Fm$$

##### Fv/Fm (TR/ABS or φP_o_) maximum quantum yield of primary PSII photochemistry

The maximum quantum yield of primary PSII photochemistry was calculated as per Strasser et al. 1995^19^.

9 $$\phi Po=1-\left(\frac{Fo}{Fm}\right)$$

Plant Efficiency Analyzer was used for Chlorophyll *a* fluorescence measurement of after 12 h of dye solution treatment on duckweed frond. Before measurement, duckweed fronds were dark-adapted for 45–60 min at 26 °C. A software Biolyzer v.3.06 (developed by Laboratory of Bioenergetics, University of Geneva, Switzerland) was used for analyzing the signals of Chl *a* fluorescence.

#### Enzyme extraction and assay

After the decolorization experiment (12 h) *Lemna* fronds treated with 15 and 30 mg/l BG4 were removed from the solution respectively. About 300 mg (fresh weight) fronds were homogenized in 5 ml ice-cold potassium phosphate buffer (0.1 M, pH 7.8) for preparing enzyme extract. The homogenate was centrifuged at 15,000 × *g* (4 °C) for 20 min (REMI, India). the supernatant was saved and used as the enzyme extract. All the preparation for enzyme extract was carried out at 4 °C and the enzyme activity was expressed in the term of U mg^−1^ fresh weight.

The SOD activity was determined spectrophotometrically by measuring its ability to inhibit the photochemical reduction of Nitro Blue Tetrazolium (NBT) at 560 nm^28^. The reaction mixture containing 100 µl L- methionine, 100 µl NBT, 10 µl riboflavin, and 100 µl enzyme extract and 2.7 ml Na_2_CO_3_ (0.05 M). The tubes containing reaction mixture were placed below white fluorescent light for 10 min after that the reaction stopped by placing the tubes in dark for 8 min and absorbance was measured at 560 nm. SOD enzyme to produce a 50% inhibition of the reduction of NBT was expressed as one unit of SOD enzyme activity.

The CAT activity was measured by the consumption of H_2_O_2_ at 240 nm^29^. The reaction mixture containing 120 µl enzyme extract, 80 µl H_2_O_2_ (500 mM) and 2.8 ml potassium phosphate buffer (50 mM). One unit of catalase activity was defined as the amount of enzyme necessary for reduction 1 mM of H_2_O_2_ per minute.

GPOD activity was determined spectrophotometrically by measuring changes in absorbance at 436 nm for 15 s. up to 5 min^30^. Reaction mixture contained 1 ml guaiacol (1%), 1.7 ml phosphate buffer (0.05 M, pH 7.0) and the reaction was initiated by adding H_2_O_2_. The enzyme required for the transformation of the substrate in 1 min is expressed as unit enzyme activity.

#### Dye removal experiment

The decolorization experiments were performed in the 250 ml beaker containing 200 ml solution of malachite green dye (Fig. 1b). Details about chemical structure and characterization of BG4 are given in Table 4. After every regular treatment interval, the sample was isolated and the remaining dye was determined with a UV spectrophotometer (ANALYTIKJENA SPECORD 200, Germany) at maximum absorbance wavelength (λ_max_) = 619 nm. A linear correlation was established between the concentration of dye and the absorbance (A) at 619 nm (λ_max_). in the range of C_dye_ = 0 to 30 mg/l. Percent dye removal was calculated by using Eq. (10).

**Table 4:** Chemical structure and characterization of BG4.

Chemical structure	
C.I. name	C.I. Basic green 4
Molecular formula	C_23_H_26_N_2_O.HCl
C.I. number	42,000
M_W_ (g/mol)	382.9
λ_max_ (nm)	619

10 $${\% }Dye removal=\left[1-\left(\frac{A}{Ao}\right)\right]\cdot 100$$

To evaluate the effects of environmental factors and operational parameters on the efficiency of dye removal, the decolorization experiments were carried out with pH values (2.0–9.0), temperature (10–50 C) and weights of plant (2.0–10 g) respectively. The pH of the dye solution was adjusted using 1 N NaOH and 1 N HCl and was measured by pH meter (Hanna HI98100, United States). To determine the reusability of *L. gibba* repeated-batch processes were performed to remove BG4^31^. When the decolorization process was completed, the same plants were used to decolorize another dye solution containing 30 mg/L BG4. This process was repeated four times.

#### FT-IR analysis

Fourier Transform Infrared (FT-IR) spectroscopy was performed according Khataee et al. (2010) by using BRUKER TENSOR 27 spectrometer, Germany^15^. For FT-IR analysis, biological treatment process was performed with 250 ml solution containing 10 mg/l of MG and 4 g of duckweed fronds. At the reaction times of 0 h (control), 6 h and 12 h samples were taken and the biological degradation products were extracted with 30 ml of diethyl ether in three times, then crystallized and used for analysis.

### Statistical analysis

The data analysis was done using SPSS (v. 21.0) software and the graphs were prepared using Microsoft office. All values presented in the paper are means of three independent replicates. Statistical analyses of data were carried out by One-way ANOVA tests and significant differences were established by Tukey (HSD) tests at *p* ≤ 0.05.

## Results and discussion

### Photosynthetic performance

**Figure 2:**
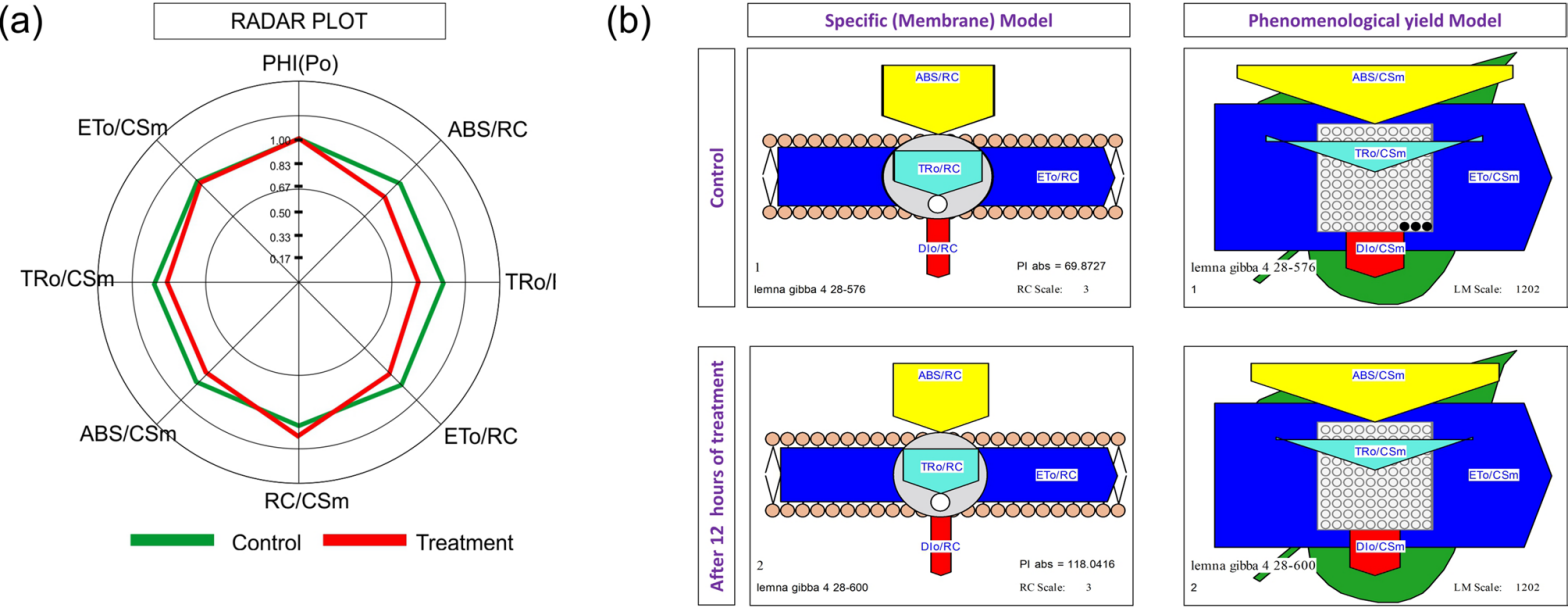
**(a)** Radar plot showing the specific, phenomenological, and Fv/Fm before and after maximum decolorization of BG4 in *L. gibba*, **(b)** specific membrane models and phenomenological yield models representing the changes in various photosynthetic parameters in control and BG4 treated *L. gibba.* The figures are created by the software Biolyzer (The JIP-test analyzing program) version 3.06 HP Jan. 2002, developed by Laboratory of Bioenergetics, University of Geneva, Switzerland.

*L. gibba* exhibited no profound effect on photosynthetic efficiency during the decolorization process. However, the activities of specific fluxes (ABS/RC, TR/RC, and ET/RC) were found more sensitive to BG4 (Table 5). The BG4-induced decline in ABS/RC and TR/RC 19.19% and 17.96% respectively. Similarly, the ET/RC reduced 9.62% during the complete decolorization of BG4. To compensate for the BG4-induced reduction in specific fluxes, the plants increased the RC/CS. The density of active RCs increased by 6.88% in BG4-treated plants as compared to controls (Fig. 2a). Transformation of inactive PSII RCs into active form displays physiological adaptation in *L. gibba* against BG4-induced chemical stress^25^.

**Table 5:** Chl *a* fluorescence parameter of *L. gibba* grow under pre- and post- BG4 treatment.

	PHI(Po)	ABS/RC	TRo/RC	ETo/RC	RC/CSm	ABS/CSm	TRo/CSm	ETo/CSm
Control	0.79 ± 0.004	1.42 ± 0.002	1.12 ± 0.007	0.81 ± 0.008	844.70 ± 1.529	1205 ± 3.606	945.85 ± 3.343	687.84 ± 2.263
After 12 h	0.80 ± 0.0005	1.20 ± 0.001	0.95 ± 0.002	0.74 ± 0.001	910.36 ± 1.452	1078 ± 2.646	866.03 ± 2.524	672.66 ± 3.045

Values are presented in the average of triplicates ± SD.

BG4 treated plants exhibited a slight reduction in light-harvesting and trapping efficiencies per cross-section (ABS/CS and TR/CS) when matched with controls. The values of ABS/CS and TR/CS decreased 10.98 and 9.83 in plants subjected to BG4 decolorization. Reduction in ABS/CS and TR/CS may be due to the decline in ABS/RC and TR/RC^32^.

Overall electron transport rate per cross-section (ET/CS) remained unchanged in duckweed during decolorization of BG4, which indicated that *L. gibba* enhanced the concentrations of active PSII RCs to maintain the rate of ET/CS. Similarly, no significant variations in Fv/Fm were observed during the entire duration (12 h) of BG4 decolorization, which indicates that *L. gibba* has high potential to maintain its photosynthetic efficiency even during/after the decolorization of BG4 by modulating the specific, phenomenological fluxes, the density of active PSII RCs and Fv/Fm (Fig. 2b). Chlorophyll fluorescence analysis demonstrates that *L. gibba* has high physiological adaption to sustain overall photosynthesis during the post-decolorization of BG4.

### Tolerance to the dye (antioxidative enzymes)

Plant enzymes play a crucial role in the biodecolorization of pollutants^13^ and directly participate in the decolorization of synthetic dyes^33^.

**Figure 3:**
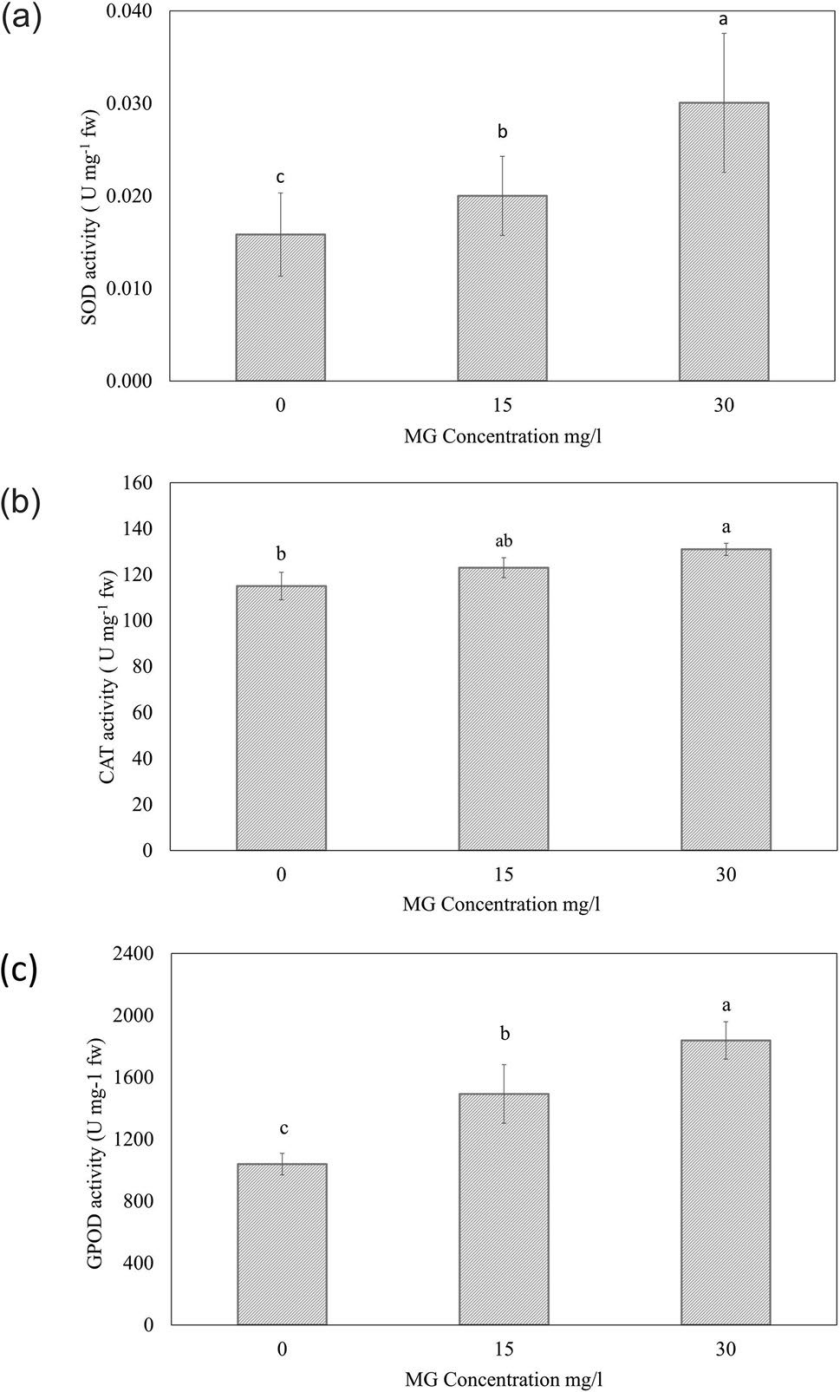
**(a)** SOD activity, **(b)** CAT activity and **(c)** GPOD activity in control and treated *L gibba* with 15 and 30 mg/l BG4. Values are presented in the average of triplicates ± SD. Different characters indicate significant differences among the results (*p* ≤ 0.05).

SOD is an important enzyme of plant antioxidant defense system and it converts two superoxide radicals (O_2_^.-^) to water and O_2_. Subsequently, the products of SOD were furthers detoxified by other enzymes CAT and GPOD and convert into less toxic compounds^34^. After 24 h of biodecolorization process, a significant induction (116.67% and 164.76%) in SOD activity was observed in *L. gibba* treated with 15 and 30 mg/l of BG4, respectively (Fig. 3a). The activity of GPOD and CAT were also increased 120.45% and 106.96% respectively from control with exposure by 15 mg/l of BG4. A significant increase was observed in GPOD and CAT activity with exposure with 30 mg/l of BG4 after 24 h (143.66% and 113.91% respectively from control) (Fig. 3b,c). As displayed in Fig. 3 antioxidant enzyme activity increases significantly (*p* ≤ *0.05*). The presence of pollutants in the environment around the plants leads to oxidative stress and produces a high amount of reactive oxygen species (ROS) and perhaps these antioxidant enzymes directly involved in the conversion of these harmful ROS into a less toxic product^35^. The amount of ROS increased due to high concentration of dye^31^ which activates the antioxidant system to protect the plant from these deleterious components^34^. Enzymes SOD, GPOD, and CAT are major components of the defense system of the plant against stress^33^.

### The effects of different parameters on removal rate

#### Effect of duckweed biomass on dye removal rate

**Figure 4:**
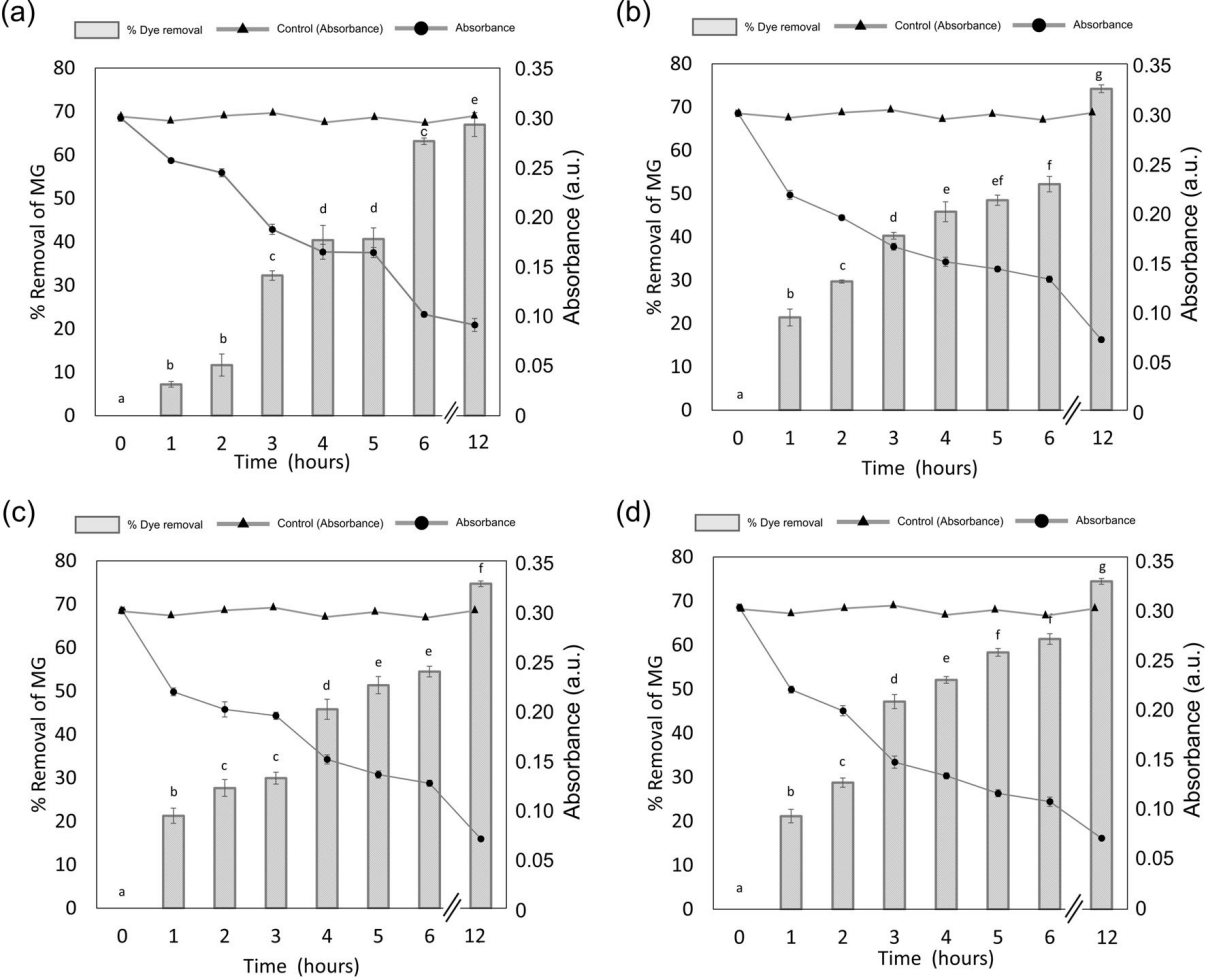
The absorbance and Percent removal of BG4 of **(a)** 2 g, **(b)** 4 g, **(c)** 6 g and **(d)** 8 g duckweed after water contaminated with 30 mg/l of BG4 dye was treated with *L. gibba* for 12 h. Values are presented in the average of triplicates ± SD. Different characters indicate significant differences among the results (*p* ≤ 0.05).

**Figure 5:**
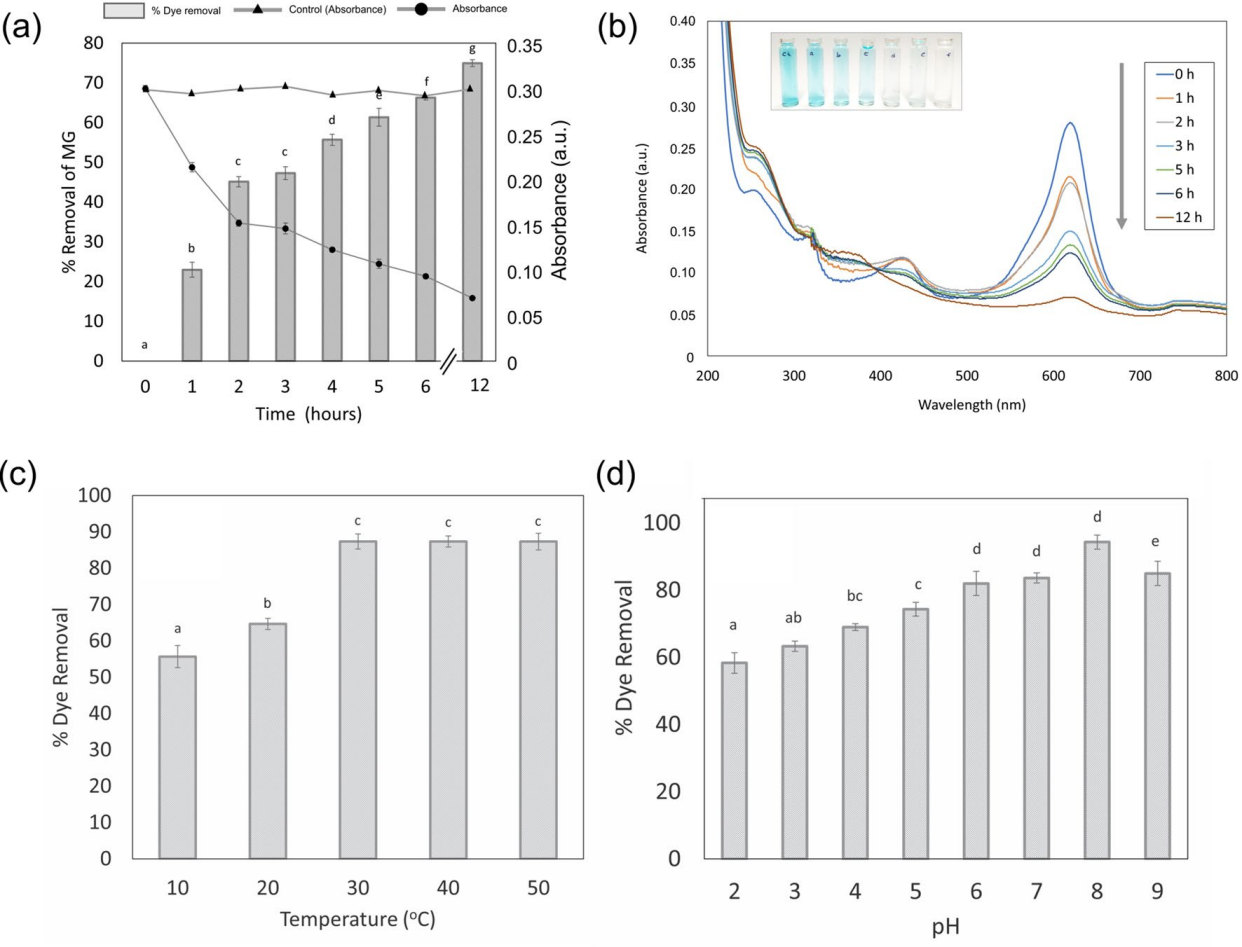
**(a)** The absorbance and Percent removal of BG4 of 10 g duckweed after water contaminated with 30 mg/l of BG4 dye was treated with *L. gibba* for 12 h. Values are presented in the average of triplicates ± SD. Different characters indicate significant differences among the results (*p* ≤ 0.05), **(b)** UV spectra of BG4 (30 mg/l) decolorization by *L. gibba* at time 0–12 h, **(c)** effect of different temperature on biodecolorization of BG4 (pH  8.0, Plant weight = 4 g, [BG4]_0_ = 30 mg/l, (**d) **effect of pH of dye solution on biodecolorization of BG4 (T = 30 °C, Plant weight = 6 g, Time 7 h).

The initial concentration of dye, temperature, and pH were kept constant in order to make a comparative study for biogenic decolorization of BG4 dye in the presence of different amounts of duckweed (2–10 g). The dye removal efficiency was significantly increased with increasing the biomass (Figs. 4, 5a) until it reached a value of 74.72% with the biomass of 6 g. The decolorization of BG4 was spectrophotometrically analyzed and the UV spectra were shown in (Fig. 5b). These results indicate that the duckweed biomass of 6 g would be the minimum desired biomass for removing dye from contaminated water (Fig. 4c). Increases in duckweed biomass provided more surface area for absorption of the dye molecule^10,31^. Another possibility of our finding is that high initial concentration of dye (30 mg/L) will provide high probability to contact between dye molecules and plant surface areas. Daneshvar et al. (2007) and Khataee et al. (2009) reported that increased initial concentration of pollutants gives results in high rate of decolorization^36,37^.

#### Effects of temperature and pH on decolorization potentials:

The temperature is an important environmental factor that alters various biological processes. The effect of temperature on biological decolorization process is one of the important and effective parameters. To determine the effect of temperature on biological decolorization was studied at the range of 10–50 °C at an initial concentration of 30 ml/l. As displayed in (Fig. 5c), high temperature triggers the removal rate of dye and results showed that the thermal deactivation of dye decolorization was not observed. The reaction of biosorption between duckweed and BG4 was an endothermic process prove through the above finding. The results are similar to literature information that high temperature induces biological dye decolorization capacity^36,38^.

Biological decolorization of dye using plants is highly pH dependent^39^. In the present study, biodecolorization of BG4 was analyzed over a range from 1.0 to 9.0 pH. It was observed that dye decolorization efficiency increases as the pH of the solution increase up to 8 (Fig. 5d). It can be understood by the concept of zero-point discharge for biomass. An isoelectric point of around 3–4 pH is determined for plant biomass^38,40^. The plant surface charged positively in acidic solution and negatively in alkaline solution. However, BG4 is a cationic dye and the high pH solution enhances the bio-adsorption of dye, hence the dye removal potential increases as reported by Vasanth et al.^16^.

#### FT-IR analysis

**Figure 6:**
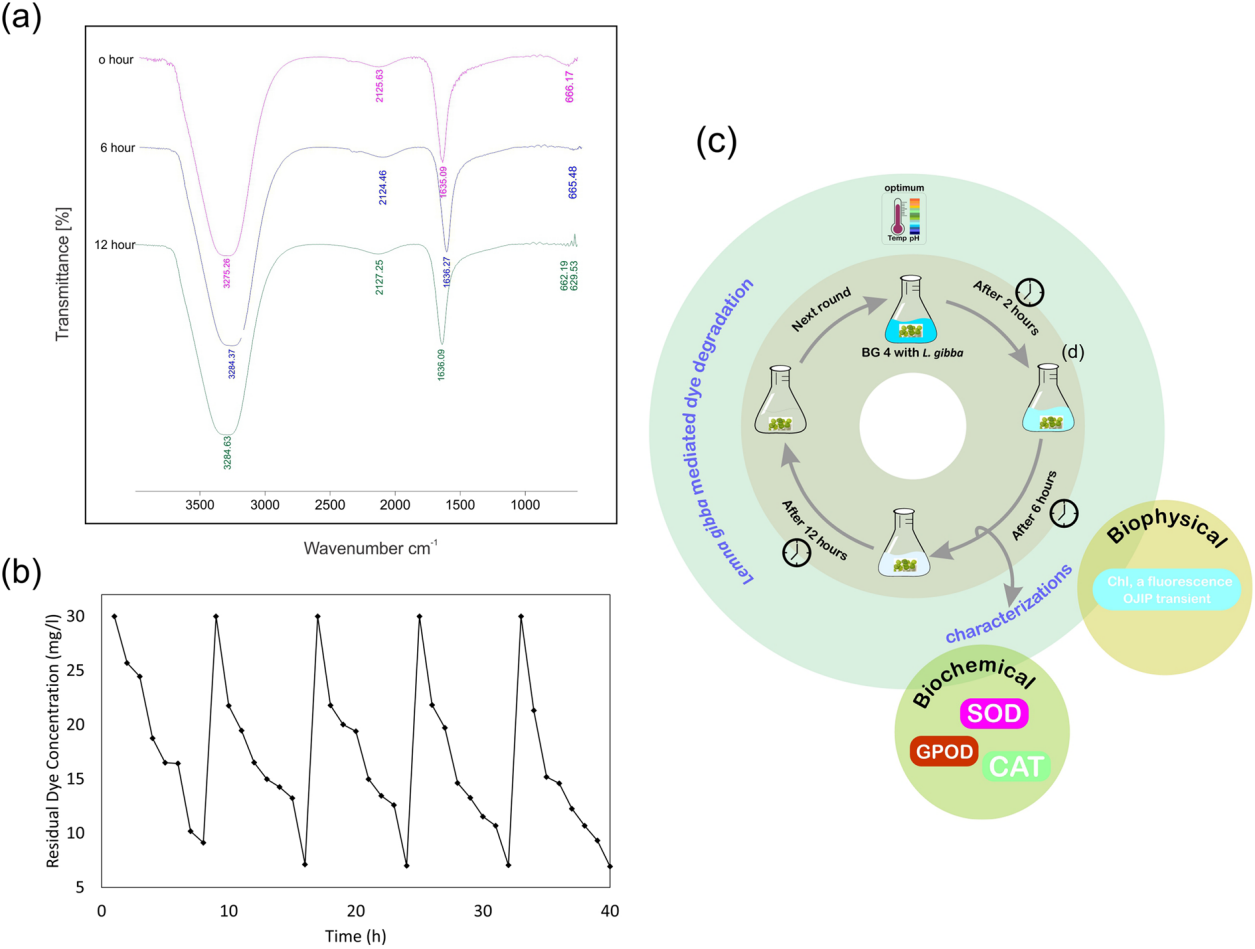
**(a)** FT-IR spectra of BG4 (10 mg/l) at times: 0,6 and 12 h (T = 30 °C, pH 8, Plant weight = 4 g), **(b)** reusability and decolorization potential of *L. gibba* to BG4 upto 5 repeated-batch operation (T = 30 °C, pH  8.0, [BG4]_0_ = 30 mg/l, Plants weight = 6 g), (**c)** over view of *L. gibba* mediated BG4 decolorization (The Figure was prepared using CorelDRAW Graphics Suite X7 Corel Corporation (Version: 17.5.0.907) https://www.coreldraw.com.

Figure 6a illustrates FT-IR spectra of BG4 treated by *L. gibba* at different reaction times. FT-IR spectrum of Malachite Green before removal showed the specific peaks in fingerprint region (2500–500 cm^−1^) benzene rings which is supporting to the peak at 1635 cm^-1^ for the C––C stretching of the benzene ring^41^. FT-IR spectrum of extracted product at 6 and 12 h reaction time displayed no significant alteration in fingerprint region which indicated that the mechanism involved in decolorization of BG4 by *L. gibba* is bio accumulation and not biodegradation. FT-IR studies are helpful in understanding the mechanism of remediation of BG4 by *L. gibba*. With the help of FT-IR studies, it can be concluded that *L. gibba* initially adsorbed the dye on its surface through electrostatic and hydrogen bond interactions among functional groups of *L. gibba* and BG4 dye. Similar results were found by Mahajan and Kaushal (2020) during the removal azo dye methyl red (MR) by macroalgae *Chara vulgaris* L^42^. However, further studies are required, which throws light on the plant’s enzymatic mechanism for decolorization of BG4.

#### Recyclability of live *L. gibba*

To examine the reusability of *L. gibba* in Basic Green 4 decolorization a repeated batch operation was performed. During five repeated batch run it was recorded that *L. gibba* showed equal dye decolorization efficiency (Fig. 6b). No adverse effect of BG4 on biomass was observed upto 5 times reusability of plants. From these results, we can conclude that *L. gibba* possesses a great ability to recycle or reusability in repetitive decolorization processes. The results also indicate that the removal of the BG4 by the duckweed is a biological decolorization process. Earlier studies demonstrate that *L. minor*^43^ and *Chara*^15^ degrade dyes AB92 and BG4 respectively. In contrast, present findings reveal that *L. gibba* has high potential of taking up BG4 and can be used as a potent biological tool to remove BG4 from polluted water.

## Conclusion

The results from present research work give a positive sign that *L. gibba* has remarkable potential for decolorization of BG4. Chlorophyll fluorescence analysis reveals that photosynthetic apparatus of *L. gibba* is highly tolerant to BG4. Plants photosynthetic efficiency did not alter even during and after the dye decolorization. BG4 treatment to *L. gibba* leads to activation of antioxidant activity which determined by the increased value of SOD, CAT, and GPOD which usually activated when plants suffering unfavorable environmental conditions. The *L. gibba* mediated BG4 decolorization depends on various parameters that were assessed in this study. As increasing pH, temperature, contact time, and plant weight the BG4 decolorization capacity was also increased. The study revealed that the temperature at 25–30 °C and pH 8.0 are considered as optimum for the best results. The repeated batch experiment confirms the reusability of *L. gibba* for BG4 decolorization. The FT-IR spectra of BG4 solution during biological decolorization under the optimized conditions revealed that this process is bioaccumulation. The overall results of the present findings highlight that duckweed *L. gibba* can be used as an effective organism for biodecolorization of BG4 thus, protecting the Earth’s hydrosphere. Figure 6c shows the over view of *L. gibba* mediated BG4 decolorization.
